# Importance of Multimodality Cardiac Imaging in the Diagnosis of Lipomatous Hypertrophy of the Interatrial Septum—A View beyond Standard Situations

**DOI:** 10.3390/life14040514

**Published:** 2024-04-16

**Authors:** Raluca Șoșdean, Mihai-Andrei Lazăr, Silvius Alexandru Pescariu, Monica-Nicoleta Mircea, Radu Ioan Lala, Cristian Mornoș, Constantin Tudor Luca, Adina Ionac

**Affiliations:** 1 Department VI—Cardiology, “Victor Babeș” University of Medicine and Pharmacy of Timișoara, 300041 Timișoara, Romania; sosdean.raluca@umft.ro (R.Ș.); lazar.mihai@umft.ro (M.-A.L.); mornos.cristian@umft.ro (C.M.); constantin.luca@umft.ro (C.T.L.), adina.ionac@gmail.com (A.I.); 2 Institute of Cardiovascular Diseases, 300310 Timișoara, Romania; mircea.monica@yahoo.ro; 3 Department of Cardiology, Faculty of Medicine, Arad Emergency County Hospital, Arad Western University “Vasile Goldis”, 310025 Arad, Romania; radu_lala@yahoo.com

**Keywords:** interatrial septum, lipomatous hypertrophy, “hourglass” appearance, multimodality imaging

## Abstract

Lipomatous hypertrophy of the interatrial septum (LHIAS) represents a benign proliferation of lipoid cells at the level of the interatrial septum (IAS) inducing an important thickening of this structure. It respects the fossa ovalis (FO) region, having a typical “hourglass” echocardiographic appearance. There are certain cases though, with unusual appearances and/or with associated pathologies that may induce similar lesions in the heart, in which the differential diagnosis cannot be guaranteed using only the standard methods. The final diagnosis has important implications in these patients’ treatment plan. In this paper, we present an unusual case of a female patient undergoing chemotherapy for lung carcinoma, suspected of right atrial thrombosis/metastasis. As the diagnosis was unclear after transthoracic echocardiography (TTE), inducing the suspicion of an IAS mass with atrial wall infiltration, bi- and tridimensional transesophageal echocardiography (TOE) was performed, revealing a severely and homogenously hypertrophied IAS respecting the FO, but lacking a clear visualization of the atrial wall. The diagnosis of LHIAS was established by cardiac magnetic resonance (CMR) that certified the adipose nature of the structure, excluding the need for invasive investigations and/or treatment options. Multimodality imaging is very important for the clinician in adopting the best management plan for each individual patient.

## 1. Introduction

The interatrial septum (IAS) is the fibromuscular structure that divides the two upper chambers of the heart, the left and the right atria. When intact, it prevents oxygenated and unoxygenated blood from mixing. The IAS is more than just a simple separating membrane. It has two main parts—the ostium primum and the ostium secundum—according to the stages of its embryological development, between the first and the second month of life. First, the septum primum develops as a crescent-shaped tissue originating posteriorly, leaving a communication between the two developing upper chambers called the ostium primum and the ostium secundum. This communication is important as the lungs are non-functional during intrauterine life. The septum secundum develops anteriorly as a thick muscular structure on the right side of the septum primum, reducing the ostium secundum to a small communication—the foramen ovalis. The ostium primum also progressively diminishes in size until it only forms a flap that covers the foramen ovalis, forming the fossa ovalis (FO). During this embryological development of the IAS, with infoldings of the roof and upper walls of the primary unique chamber, mesodermal tissue can be entrapped in the primitive septum, forming areas of adipose tissue. Later, these areas may expand and even bulge in the atrial cavity, thus causing the lipomatous hypertrophy of the interatrial septum. This process mainly involves the septum secundum, and thus the bulging will become more pronounced in the right atrium, respecting the fossa ovalis. Although uncommon, adipose hypertrophy may also involve the atrial walls [1,2,3,4].

LHIS consists of a nonencapsulated fat deposit within the IAS. It is mainly composed of hyperplastic adipose cells including both white and brown adipose tissue as well as atypical and sometimes hypertrophied cardiomyocytes. The role of brown adipose tissue at this level is still unknown. Going by the definition, hypertrophy is present beyond the value of 20 mm, sometimes reaching 30 mm while respecting the FO [5,6,7,8,9]. Usually, it is related to advanced age, obesity, and female gender, and it is a benign entity [2,10]. When reaching significant proportions, it may be misdiagnosed if not investigated thoroughly, putting the patient at risk of unnecessary invasive/surgical treatment or diagnostic procedures. It can be mistaken with a variety of other entities like lipomas, lymphoma, myxoma, rhabdomyoma, mesothelioma, and fibroma/fibroelastoma [4,11]. This usually occurs when the appearance is non-characteristic and/or it cannot be investigated properly through echocardiography, the most commonly used cardiac imaging technique. Also, there are patients with certain pathologies, like neoplasms, that warrant a more systematic and thorough investigation, in order to exclude possible effects on the heart (for example, thrombosis and metastases). Nonetheless, a correct diagnosis and description are valuable in individuals that undergo percutaneous interventions that imply transseptal puncture [1,4].

Although exhibiting a similar adipose composition, LHIS is different from cardiac lipoma, a tumor with a significantly lower incidence. Unlike LHIS, lipoma is encapsulated, and it can grow anywhere within the heart. When attached to the IAS, it usually has a wide insertion and protrudes in the atrial cavity (usually left). When located inside the atrial cavity, it has a hyperechogenic and homogenous aspect [7,12].

Normally, LHIS is an asymptomatic entity; however, if it grows to significant proportions, it can compress surrounding structures like the vena cava, producing venous flow obstruction [11]. It may also be associated with arrhythmias, syncope, and heart failure signs and symptoms. The infiltrated septum may predispose to atrial re-entry arrhythmias but also to conduction abnormalities. Various amounts of white and brown fat may cause inflammation and even fibrosis which disrupts the muscle fibers [13]. Usually, LHIS requires no treatment, but when symptomatic, surgical excision may be an option [4].

Echocardiography, transthoracic and/or transesophageal, can describe the typical appearance of the hypertrophied IAS respecting the FO and may offer hints about tissue characterization such as the lack of vascularization by using color Doppler and/or contrast. Cardiac CT and/or MRI provides a better tissue characterization with a high fat specificity, making tissue biopsy unnecessary in these cases. Sometimes, it may even be incidentally diagnosed during positron emission tomography scans [7,9].

In this paper, we present a special clinical case in which we considered it important to use multimodality imaging for the diagnosis to be accurate, as it had important implications for the patient’s correct management. The diagnostic work-up and the imaging techniques that bring important information in this regard are thoroughly discussed.

## 2. Clinical Case Presentation

In what follows, we present the case of a 51-year-old female who was known with grade II arterial hypertension, type 2 diabetes mellitus, and dyslipidemia, for which she received the corresponding treatment with angiotensin 2 receptor 1 blocker, beta-blocker, statin, fibrate and oral antidiabetic drugs, and hereditary thrombophilia (heterozygous factor V Leiden mutation, PAI-1 4G/5G and AGT M235T mutations, and homozygous MTHFR A1298C mutation). She was diagnosed 18 months before presenting to our clinic with right superior lobe (RSL) pulmonary adenocarcinoma with bilateral dissemination and left lumbar metastasis (stage IV, TNM cT2a cN2 cM1). She was started on chemotherapy through a central venous catheter with antifolate, pemetrexed; a platinum-containing compound, carboplatin; and an immune checkpoint inhibitor, pembrolizumab; along with corticosteroid, dexamethasone; antihistamine, Symphoral; and histamine H2 receptor antagonist, famotidine, as adjunctive therapy. Nine months later, she was diagnosed with catheter-induced right cephalic vein thrombosis which resolved after initiation of anticoagulant treatment. The evolution of the neoplastic lesions was favorable at the beginning, with a reduction in the RSL tumor, disseminated pulmonary micronodules, mediastinal adenopathy, and lumbar tumor, followed by a stagnation but with no supplementary lesions.

The patient presented to our clinic with the suspicion of cardiac metastasis and/or right intra-atrial catheter-related thrombosis, after the discovery of a mass inside the right atrium which did not respond to anticoagulation therapy.

**Figure 1:**
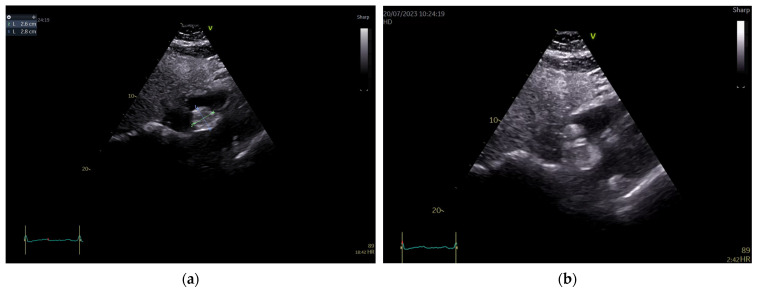
Transthoracic echocardiography, subcostal views. A round-oval, apparently homogenous mass attached to the IAS is visualized bulging in the RA. (**a**) Measurement of the mass revealed diameters of 26/28 mm. (**b**) There is no clear delineation of the RA-free walls and an infiltration at this level cannot be excluded.

The patient’s standard blood tests as well as their resting electrocardiogram were within normal limits. Transthoracic echocardiography showed a normal-sized left ventricle with preserved ejection fraction (57% by tridimensional assessment) and a global longitudinal strain of −20.5%, as well as a normal-sized right ventricle with an ejection fraction within normal limits (50% by tridimensional assessment), thus demonstrating a non-significant cardiotoxic effect of chemotherapy until this moment. There was a mild degenerative mitral regurgitation associated with a mild functional tricuspid regurgitation, without any other valvular problems. In the apical four chamber and subcostal views though, a round-oval-shaped mass was identified in the right atrium apparently attached to the atrioventricular pole of the interatrial septum in a sessile manner. Pericardiac fat seemed well represented. The mass looked iso-hyperechogenic, slightly inhomogeneous, mobile with the IAS, and measured 28/26 mm. At first glance, it had an appearance similar to a myxoma. There was also a thickening of the RA wall detected (artefact? impregnation?) (Figure 1).

**Figure 2:**
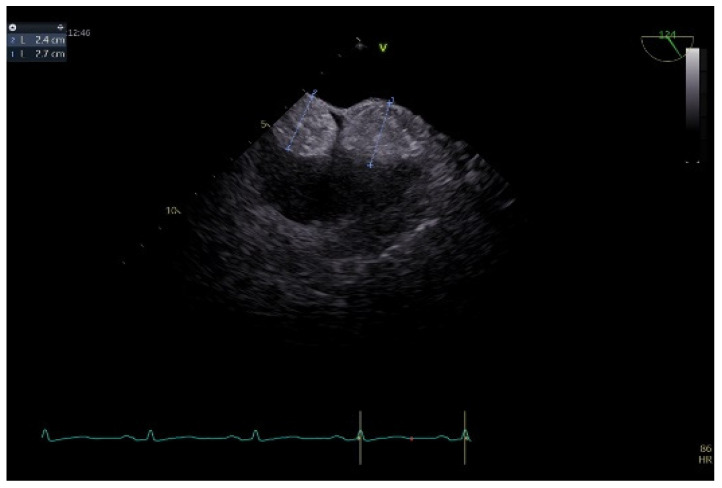
Bidimensional transesophageal echocardiography, modified bicaval view. Two so-called sessile masses (24–27 mm), at the level of the IAS respecting the fossa ovalis, with the appearance of separate structures.

**Figure 3:**
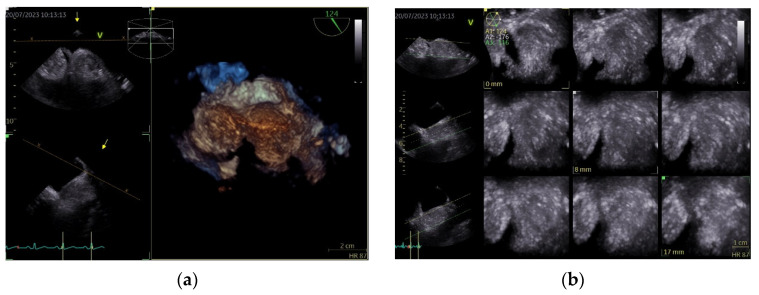
Transesophageal echocardiography, tridimensional reconstruction. (**a**) Four-dimensional zoom acquisition revealing two so-called masses attached to the IAS with the appearance of distinct structures, bulging in the RA (inferior) and imprinting the LA surface of the IAS (superior); (**b**) 4D zoom acquisition after multislice analysis, confirming the homogenous aspect of the “structures”.

To better describe it, a transesophageal echocardiography was performed. We were surprised to see a second similar so-called mass at the superior pole of the IAS, with the same aspect as described with transthoracic echo. In fact, the IAS was intensely thickened in a diffuse manner except for the fossa ovalis and had the appearance of an “hourglass”, suggesting a possible lipomatous hypertrophy of the interatrial septum. Using both bidimensional (Figure 2) and tridimensional echo (Figure 3), the masses seemed to be encapsulated, homogenous, and attached to the IAS while clearly not being a part of it, aspects that raised some questions in this special patient. There was no color Doppler signal inside the masses, suggesting a lack of vascularization. They lacked any contact and/or continuity with the cardiac valves, an aspect that helped in the differential diagnosis with certain tumors originating on these structures. The right atrial walls were not clearly and entirely viewed. Also, the fact that these masses were not described during previous evaluations was quite unusual. The appearance did not resemble that of a thrombus, since even if the patient had thrombophilia, she was well anticoagulated, and the position and shape of the “masses” did not resemble thrombi. A malignant tumor/metastasis should have been more invasive, irregular, and not respecting the fossa ovalis. The echocardiographic structure of the masses could have resembled a cardiac myxoma though, but possessing two such masses respecting the FO was uncommon. The same situation applies to other possible benign tumors and/or endocarditis. A possible abscess in the context of endocarditis should have also yielded high-inflammation markers. Cardiac amyloidosis was excluded since it did not spare the FO and there were also no other signs of this pathology. Papillary fibroelastoma is usually attached to heart valves, and hemangioma can be found anywhere, most frequently in the LV and RV but also in the RA. Rhabdomyomas are usually found in the ventricular walls and valves, in infants and children. Fibroma is most frequently found in the ventricles, in infants, children, and young adults. Myxoma is normally diagnosed between 30 and 60 years of age, or even earlier if part of Carney syndrome. It can be located anywhere in the heart, but most often within the IAS, pediculated and protruding in the LA. However, a myxoma is a gelatinous non-homogenous mass, due to areas of necrosis and/or hemorrhage, and it sometimes has a villous surface, characteristics that helped exclude this pathology. There are usually multiple malignant tumors which can also be found in any part of the heart. They are irregular masses with an extensive/invasive nature. The most frequent is angiosarcoma which is often located in the RA. Metastatic tumors reach the heart either by direct extension, lymphatic and hematogenous extension, or intracavitary extension through the vena cava and/or pulmonary veins. They are most often secondary disseminations from lung cancer (men) and/or breast cancer (women) [3,7,8,14].

**Figure 4:**
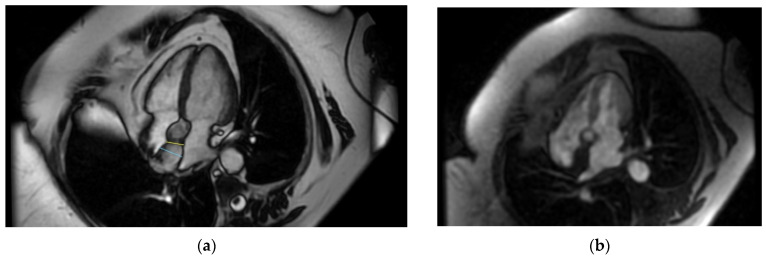
Cardiac MRI. (**a**) Four-chamber-view, steady-state-free precession image revealing interatrial septal hypertrophy—25 mm with “chemical shift”; (**b**) perfusion image—no caption of contrast at first-pass contrast.

**Figure 5:**
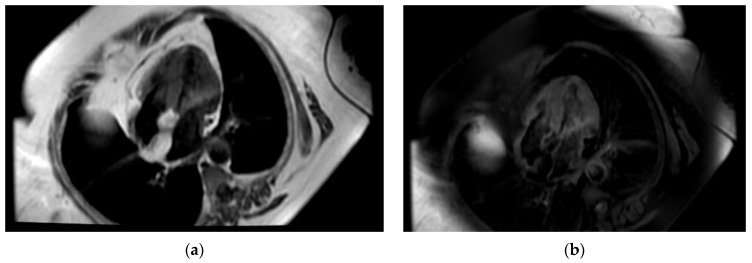
Cardiac MRI. (**a**) Double-inversion-recovery T1 sequence (4 chambers) revealing a hyperintense interatrial septum suggestive of fat; (**b**) double-inversion T1 sequence with fat suppression revealing a hypointense interatrial septum.

**Figure 6:**
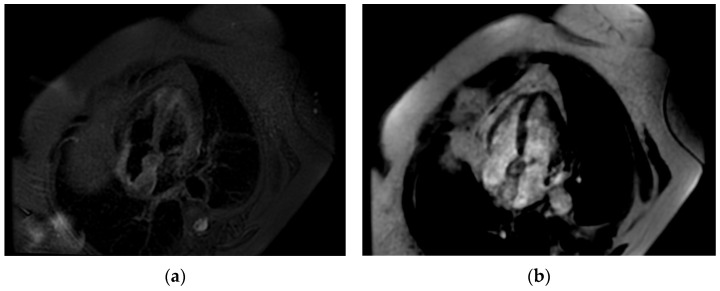
Cardiac MRI. (**a**) Triple-inversion-recovery T2 sequence of isointense interatrial septum; (**b**) late gadolinium enhancement (LGE)—enhancement in interatrial septum.

Even though the diagnosis was suspected, a cardiac magnetic resonance was performed in order to be accurate, given the implications a wrong diagnosis might have had. The “hourglass” aspect of the IAS with significant hypertrophy was confirmed in the cine-balanced steady-state-free precession (bSSFP) sequences. There was no contrast up-take at this level, suggesting a lack of vascularization, and the structure showed a high signal in T1 and a low/isointense signal in T2 with a very similar aspect to the very well-represented pericardial and mediastinal fat (Figure 4, Figure 5 and Figure 6). The diagnosis of lipomatous hypertrophy of the interatrial septum was established.

Given that the patient had no symptoms related to this diagnosis, like syncope, obstructive syndrome, arrhythmias, and heart failure, a close follow-up was decided, without any other treatment. She was followed-up for 9 months without a significant change in her clinical status nor in the echocardiographic aspect of her IAS.

## 3. Discussion and Review of the Literature

The present case shows a patient with multiple significant pathologies which might have had induced cardiac pathologies requiring additional investigations. The hereditary thrombophilia and the history of catheter-related thrombosis raised genuine suspicions of thrombosis especially in the context of suboptimal transthoracic echocardiography images. The improved visualization of the IAS with TOE made this diagnosis highly unlikely. Given the patient’s cancer background, one cannot exclude with certainty a secondary dissemination, even in the presence of an hourglass shape of the IAS, highly suggestive for a lipomatous hypertrophy. On the other hand, it was unusual for secondary dissemination to develop exactly in the area in which chemotherapy was delivered. Still, this pathology along with other malignant or benign tumors, although less likely, could not be excluded until tissue characterization by CMR was available. CMR could distinguish very well between fat tissue and other types of tissue, demonstrating an association between the IAS aspect and structure and the high quantity of pericardial and mediastinal fat, which could not be very well quantified by echocardiography. There are several reports that demonstrate such an association as well as with obesity, which was also concordant in our case [2,10,15]. Boeriu E. et al. reported generalized lipomatosis induced by chemotherapy with platinum agents in a 16-year-old girl treated for teratoma [16]. Given the fact that our patient was unaware of this problem until now and she lacked any previous imaging results that could describe it, we even thought about a possible reaction of the IAS in relation to the chemotherapy catheter.

Regarding epidemiology and manifestations, in a prospective study including 1292 patients which were evaluated by thoracic multislice CT, Heyer C. M. et al. demonstrated an incidence of 2.2% of LHIS, with a medium thickness of 32 mm, and this was mainly associated with obesity, emphysema (64%), increased epicardial fat (75%), and ECG anomalies (62%). The fat was distributed along the coronary sinus and anteriorly along the lateral wall of the aorta and the wall of the right atrium. Even since then, it was suspected that the involvement of the atrial wall may change/distort the architecture of the cardiac myocytes and impede the normal conduction of the electrical impulse resulting in arrhythmias. In one patient, hemodynamic obstruction was detected [3]. In a retrospective study including 175 patients, Mallio C.A. et al. demonstrated an important association between visceral fat and LHIS. Total adipose tissue as well as visceral adipose tissue were predictors for the extent of LHIS [17].

Regarding the proper diagnosis of this entity, besides transthoracic and transesophageal echocardiography, there are also other imaging techniques that can offer important information, like computed tomography, cardiac magnetic resonance, and even positron emission tomography (PET) scans, especially where the clinical and paraclinical backgrounds raise doubts.

Echocardiography is the most widely available, cost-effective, and easy-to-use imaging technique. It can easily reveal the typical aspect of the IAS—hourglass and/or dumbbell—when lipomatous hypertrophy is present, especially when the image is optimal. There are several situations though in which the image is suboptimal, and the depicted aspect is not easy to diagnose, like in our case, in which a “single mass” aspect attached to the IAS was detected by transthoracic echo. This may be due to a positional suboptimal visualization of the IAS or to a scarce echogenicity of the patient for various reasons (obesity, emphysema, etc.) [2,18]. Several authors reported the same suboptimal defined aspect, requiring a better visualization of the IAS. Licordari R. et al. demonstrated a right atrial mass with no clear definition in a 55-year-old patient also suffering from hypertension, obesity, and subcutaneous lipomatosis [5]. Lampropoulous K.M. et al. described a lipomatous membrane separating the two atria in a 65-year-old patient with atrial fibrillation, complaining of palpitations [2]. Papaetis G.S. et al. described by TTE a mass at the base of the RA originating from the IAS in a 73-year-old female patient with paroxysmal atrial fibrillation and symptoms of heart failure, in which, also by TOE, a large sessile mass attached to the IAS respecting the FO was described [19]. She underwent resection of the mass and IAS reconstruction, and the histopathological evaluation revealed LHIS. Joseph G. et al. also described a mass in the RA by TTE in a 67-year-old female which presented with supraventricular paroxysmal tachycardia. The mass was confirmed by TOE, excluding interference with other structures, and there was a high suspicion of cardiac myxoma. The adipose nature of the mass was better demonstrated with CMR [20].

As reported by most of the authors, the next step when TTE is inconclusive is to perform a TOE given the close position of the probe to the IAS and the high frequency of the transducer, ensuring a high-quality image, and only after to continue with more complex imaging techniques. TOE also brings information about other associated pathologies, important especially when, for example, a transseptal puncture intervention is scheduled. Intracardiac echocardiography can also be used, but usually not for diagnostic purposes, but for guiding percutaneous procedures that imply the IAS, in order to avoid complications induced by the hypertrophied septum (i.e., diminished manipulation of the catheters, laborious transseptal puncture, etc.) [4]. Yu Y. et al. reported a case of LHIS with an ostium secundum atrial septal defect in a 68-year-old patient presenting with chest pain and palpitations. TTE revealed a defect with a flow ratio of 2, along with an otherwise significantly thickened IAS. TOE confirmed these findings and better described the dumbbell appearance of the IAS, with a maximal thickness of 20.7 mm. Cardiac computed tomography demonstrated a clearly defined mass at the level of IAS, smooth, without contrast enhancement, with a similar appearance to the subcutaneous fat. Perfusion imaging demonstrated no hypermetabolic lesions at the cardiac level. Guided by ICE, they managed to successfully occlude the ASD by percutaneous intervention [21].

Tridimensional echocardiography evaluation of the IAS may bring supplemental information in case of an optimal image, especially during TOE, since it reveals a spatial and complete visualization of the IAS and the infiltrative nature of the pathology. On the other hand, in our case, with a quasi-suboptimal image even with TOE, it may not bring sufficient proof for a definite diagnosis. By creating multiple slices through the structure, it can provide data regarding composition but no clear information about the tissue type. The presence of hypertrophied myocytes and different types of adipocytes may create the appearance of a pseudo-capsule, which may raise some questions of differential diagnosis, as it did in our case.

In order to better visualize tissue composition, cardiac computed tomography and/or cardiac magnetic resonance is recommended. Both can be used for definitive diagnosis, avoiding biopsy, although MRI is an expensive and less available imaging technique [13]. Biopsy carries certain risks for the patient, including hemorrhage, arrhythmias, perforation, etc., and should be avoided [20]. Joseph G. et al. demonstrated the true nature of the echocardiographically detected RA mass by performing contrast-enhanced CT and CMR with T1- and T2-weighted images including fat suppression and LGE, showing a widespread hypertrophy due to fatty infiltration [20]. On contrast-enhanced computed tomography images, LHIS is a smooth, non-enhancing, and clearly marginated mass with a similar appearance to subcutaneous and/or pericardial fat [22]. Also, Heyer C.M. et al. evaluated LHIS in their study by using multislice computed tomography and defined it as the presence of a non-enhancing fatty mass within the cardiac interatrial septum and a thickness of 20 mm [3]. Cardiac magnetic resonance describes the same aspect of a clearly defined mass, with a characteristically high-intensity signal in T1-weighted images with mild inhomogeneous late gadolinium enhancement, highlighting fat accumulation [22]. A clear differentiation between adipose and muscular tissue can be made, as both blood and adipose tissues have a high T1/T2 ratio, providing a strong signal, whereas the myocardium has a low ratio, thus providing a low signal [5]. The same aspect was described in our case. Licordari R. et al. performed CMR for their case, describing the aforementioned aspects as well as the so-called “India-ink artefact” that appears as a low signal, delineating LHIS at the fat–water interface [5].

In our patient, the computed tomography examinations that were performed did not utilize cardiac structure processing software. This is why clear images of the IAS could not be obtained even with postprocessing, in order to establish a reliable differential diagnosis. We chose not to immediately repeat this investigation, since it has the inconvenience of carrying a high degree of irradiation.

LHIS can sometimes be incidentally detected when performing a PET scan for other indications. LHIS has a variable fluoro-deoxy-glucose up-take, and this can be misleading. Chiocchi M. et al. reported a case of an 81-year-old patient with a history of aortic prosthesis and periprosthetic endocarditis in which there was an appearance of FDG up-take in the IAS. The IAS looked thickened, bilobed, and adipose-dense with a thickness of 21 cm. The correct diagnosis of LHIS versus recurrence of endocarditis was made by comparing the present study to the old one, where there was an intense/massive FDG up-take in the perivalvular area and structures due to infection, masking the focal up-take in the IAS. LHIS has a variable FDG up-take, and this is believed to be due to the variable amount of brown fat they contain. Due to this aspect, the results with the PET scan alone were frequently ambiguous and necessitated clarification by using other techniques, such as cardiac MRI. By combining the PET scan with angio-CT (PET/CT), the sensitivity and specificity increased, by better delineating the area of focal up-take in the IAS. The authors also highlight the importance of comparing the present imaging studies with previous ones if available [13].

## 4. Conclusions

Multimodality imaging is the key in establishing the correct diagnosis in patients with lipomatous hypertrophy of the interatrial septum, especially when certain misleading echocardiographic aspects are present and/or certain pathologies with cardiac impact are associated. Our case confirms that also in these patients, by using the right imaging techniques, the diagnosis can be reliably established without the need for biopsy.

## Data Availability

Data are contained within the article.
